# Reducing Postpartum Weight Retention and Improving Breastfeeding Outcomes in Overweight Women: A Pilot Randomised Controlled Trial

**DOI:** 10.3390/nu7031464

**Published:** 2015-02-25

**Authors:** Julia Martin, Lesley MacDonald-Wicks, Alexis Hure, Roger Smith, Clare E Collins

**Affiliations:** 1 School of Health Sciences, Faculty of Health and Medicine, University of Newcastle, Callaghan, Newcastle 2308, New South Wales, Australia; E-Mails: Julia.Martin@hnehealth.gov.au (J.M.); Lesley.Wicks@newcastle.edu.au (L.M.-W.); 2 School of Medicine and Public Health, Faculty of Health and Medicine, University of Newcastle, Callaghan, Newcastle 2308, New South Wales, Australia; E-Mail: Alexis.Hure@newcastle.edu.au; 3 Mothers and Babies Research Centre, Hunter Medical Research Institute, John Hunter Hospital, Level 3, Endocrinology, Locked Bag 1, Hunter Region Mail Centre, Newcastle 2310, New South Wales, Australia; E-Mail: Roger.Smith@newcastle.edu.au; 4 Priority Research Centre in Physical Activity and Nutrition, Faculty of Health and Medicine, University of Newcastle, Callaghan, Newcastle 2308, New South Wales, Australia

**Keywords:** maternal, pregnancy, dietary intake, breastfeeding, postpartum weight retention

## Abstract

Overweight and obesity is prevalent among women of reproductive age (42% BMI > 25 kg/m^2^) and parity is associated with risk of weight gain*.* Weight gain greater than that recommended by the Institute of Medicine (IOM )is also associated with lower rates of breastfeeding initiation and duration in women. The aim of this pilot randomised controlled trial is to examine the feasibility of recruiting and maintaining a cohort of pregnant women with the view of reducing postpartum weight retention and improving breastfeeding outcomes. Women (BMI of 25–35 kg/m^2^ (*n* = 36)) were recruited from the John Hunter Hospital antenatal clinic in New South Wales, Australia. Participants were stratified by BMI and randomised to one of three groups with follow-up to six months postpartum. Women received a dietary intervention with or without breastfeeding support from a lactation consultant, or were assigned to a wait-list control group where the dietary intervention was issued at three months postpartum. Feasibility and acceptability was assessed by participation rates and questionnaire. Analysis of variance and covariance was conducted to determine any differences between groups. Sixty-nine per cent of the participants were still enrolled at six months postpartum. This pilot demonstrated some difficulties in recruiting women from antenatal clinics and retaining them in the trial. Although underpowered; the results on weight; biomarkers and breastfeeding outcomes indicated improved metabolic health.

## 1. Introduction

Overweight and obesity is prevalent among women of reproductive age (25–34 years) with 42% having a BMI > 25 kg/m^2^ [1]. Having children is associated with maternal weight gain, particularly in the long-term [2]. Fifty to eighty percent of women retain 1.4–5 kg up to 12 months postpartum, with 20%–50% retaining 5 kg or more [3,4,5,6,7]. Weight gain increases the risk of developing diabetes and heart disease [8,9]. The amount of weight retained after pregnancy can shift women from the healthy weight category into the overweight or obese BMI categories. Starting the next pregnancy at a higher weight increases the risk for poor pregnancy outcomes [9,10], such as gestational diabetes (RR = 2.09 for BMI > 30 kg/m^2^) [11], delivery intervention [10], macrosomia (OR = 1.57 for BMI >25–30 kg/m^2^ and 2.36 for BMI > 30 kg/m^2^) [12,13] and lower rates of breastfeeding initiation and duration [14]. Women with a high BMI have been reported to have a 7% lower breastfeeding initiation rate and breastfeed for six weeks less on average, compared to women with a normal BMI (18.5 to 24.9 kg/m^2^) [15]. Concomitantly, evidence from a nationally representative sample of Australian women indicate that women are not eating in a manner compliant with national food group recommendations, and therefore, may not be meeting their nutrient intake targets [16].

Childbearing presents an opportunity to facilitate behaviour change towards a healthy lifestyle [17]. Recently, mothers who had a BMI of >25 kg/m^2^ were consulted to determine barriers to postpartum weight loss [18]. The 10 face-to-face interviews reported that the major barriers included a lack of time, maternal low energy levels, the low priority of weight loss, overall low motivation and psychological concerns [18]. Despite this, there is evidence to suggest that women can be motivated to make healthy choices for themselves and their families, and frequently seek advice and support from family, friends and health professionals during pregnancy and after birth [19,20]. Women at this life-stage have frequent contact with clinicians, which provide potential opportunities to implement lifestyle education.

There is growing evidence that overweight and obese women have reduced rates of breastfeeding initiation and breastfeed for a shorter duration, likely due to the physical size of the breast and diminished lactogenesis [21,22,23,24]. In a longitudinal cohort (USA, *n* = 405), overweight and obese women were 1.8 and 2.2 times more likely to have delayed lactogenesis compared to underweight and normal weight women [22]. In a similar study (*n* > 37,000), overweight and obese Danish women had a shorter breastfeeding duration compared to normal weight women (RR: 1.12, 95% CI: 1.09–1.16 for overweight and RR: 1.39, 95% CI: 1.19–1.63 for obese) [25]. Lactation adds to energy expenditure, and therefore could assist with weight loss in the postpartum period [5]. In combination, this suggests that women with a higher BMI may need to be targeted for additional breastfeeding support.

Two Cochrane systematic reviews assessed a range of antenatal and postnatal educational methods used to enhance breastfeeding initiation [26] and duration [27]. These included formal and informal education, one-on-one and group education, workshops, peer counseling, discussion groups, practical skills and were provided individually and in combination. The antenatal interventions that improved breastfeeding initiation included peer counseling and regular education from an International Board Certified Lactation Consultant (IBCLC) [26,27]. The reviewers concluded that there was a lack of well-conducted Randomised Controlled Trials (RCTs) in this area, and this research was needed to determine the best ways to improve breastfeeding initiation and duration rates, in particular with a focus on those at risk of suboptimal initiation and duration rates such as overweight and obese women.

The aim of this study was to determine the feasibility of delivering an intervention during pregnancy (at week 35), which aimed to reduce weight retention in women up to 12 months postpartum, who were overweight and obese before pregnancy and who were intending to breastfeed. While interventions for postpartum weight loss or to optimise breastfeeding have been trialed as separate interventions, to the best of the authors’ knowledge, there has not been a randomised controlled trial combining both. In the current study a dietary intervention for weight loss, with and without lactation support, was compared to a wait-list control group with patterns of breastfeeding also compared between groups. The intent on including a lactation consultant (IBCLC) was to increase the duration of breastfeeding in overweight and obese women to positively impact on energy balance.

## 2. Experimental Section

**Figure 1:**
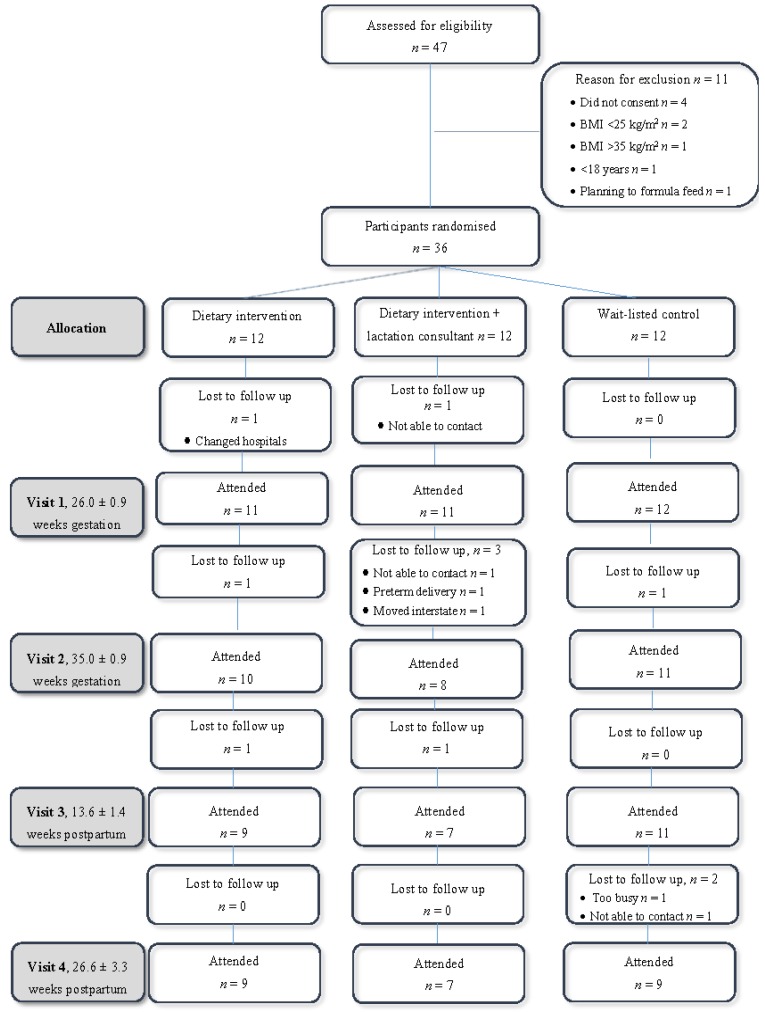
Flow diagram of recruitment and randomisation for a pilot randomised controlled trial.

A pilot, RCT was undertaken at a public tertiary obstetrics hospital in Newcastle, Australia, from October 2010 to September 2011. The approved study protocol prohibited direct recruitment by research staff, therefore flyers were distributed in clinic which encouraged potential participants to seek further information. Eligible participants were randomised to one of three groups: (i) antenatal dietary intervention for postpartum weight loss; (ii) antenatal dietary intervention and breastfeeding support for postpartum weight loss; or (iii) wait-listed control, where dietary intervention for weight loss was offered at three months postpartum. The baseline study visit was conducted at approximately 26 weeks gestation followed by a study visit at 35 weeks gestation (study visit 2) and then three months postpartum (study visit 3) as outlined in Figure 1.The fourth and final study visit was scheduled for approximately six months after birth. Antenatal visits were conducted at the John Hunter Hospital and postpartum visits were completed at the hospital or in the participants’ home if they were unable to attend the hospital. Ethics approvals were provided by the Hunter New England Health Human Research Ethics Committee and the University of Newcastle Human Research Ethics Committee.

### 2.1. Participants

The research dietitian circulated the study flyer in the obstetrics clinic (*n* = 423 flyers were distributed in the clinic by clinic administrators) any pregnant women who inquired were provided with written information about the study in the antenatal clinic by midwives and the study dietitian (JM), from October 2010 to September 2011. The inclusion criteria were: aged > 18 years, pre-pregnancy BMI 25–35 kg/m^2^, intention to breastfeed, singleton pregnancy, English-speaking, and <26 weeks gestation at the initial screening. Women also had to agree not to participate in any other weight loss program in the postpartum period while enrolled in the study. Self-reported pre-pregnancy weight and height was recorded during the initial screening and used to calculate BMI to determine if the participants met the inclusion criteria. Previous research has demonstrated self- report compared to actual pre pregnancy weight results in the same BMI classification [28].

### 2.2. Study Design

#### 2.2.1. Randomisation

Block randomisation (groups of three) using a computerised generated random number sequence was used to randomise women who were also stratified by pre-pregnancy weight status categories of overweight (BMI 25–29.99 kg/m^2^) and obesity (BMI 30–35 kg/m^2^). Numbered cards allocating women to an intervention group or the control group were placed in opaque, sequentially numbered envelopes. The person responsible for participant allocation (LMW) did not have direct contact with participants, therefore allocation concealment was maintained.

#### 2.2.2. Blinding

The study dietitian (JM) was blinded to the participant’s group allocation for women in the two dietary intervention groups. Blinding was maintained by ensuring the study dietitian was not aware of the group allocation until post study, and by asking the participants not to reveal their allocation*.* The researcher responsible for allocation concealment (LMW) contacted the study’s lactation consultant directly with the participant’s details for those allocated to the dietary intervention and breastfeeding support arm of the trial. The lactation consultant then contacted the participant directly to arrange follow up. By necessity the study dietitian was not blinded to the control group.

#### 2.2.3. Dietary Intervention

Both intervention groups received the antenatal dietary intervention for postpartum weight loss, provided by an Accredited Practicing Dietitian (APD) at 35 weeks gestation (study visit 2). Specifically, women were educated to implement the self-directed weight management program “Total Eating Management System” (TEMplate System™) post-partum. The TEMplate System™ is based on social cognitive theory and includes goal setting, cognitive restructuring, and self-monitoring of food, physical activity, and weight. Energy intake was targeted at 7000 kJ/day (standard weight loss diet of 5000 kJ plus 2000 kJ per day lactation allowance), with additional 500 kJ blocks allocated based on physical activity undertaken. Participants were provided the TEMplate System™ which is based on based on healthy eating principles and the five food groups, including the lunchbox and dinner disc for maintaining meal portion control. Education was provided to ensure participants could successfully implement the following four steps to the program:

The TEMplate System™ encourages self-efficacy and uses four steps:

1. Eat a healthy breakfast;
2. Pack a daily lunch and snack box ahead of time;
3. Use TEMplate™ dinner disc to guide portion size at main meals, including four different coloured vegetables;
4. Adjust “extras” (1 extra = 500 kJ) to balance energy expenditure from physical activity and lactation, for example women exclusively breastfeeding were given advice on adding an additional 4 extras to their eating plan, partially breastfeeding were allowed an additional 2 extra servings The APD provided advice on appropriate nutrient dense “extras” that could be added to the diet.

The Wait-List Control Group Received Standard Antenatal Care and the Dietary Intervention Was Offered at Three Months Postpartum (Study Visit 3).

#### 2.2.4. Lactation Support

One arm of the dietary intervention groups also received breastfeeding support provided by an accredited Lactation Consultant (IBCLC)*.* “The lactation consultant gave advice on lactation issues only. There was no further dietary advice provided by the lactation consultant. During the antenatal period, participants attended two 30 min face-to-face education sessions with the IBCLC to discuss the fundamental elements of breastfeeding, previous breastfeeding experience, infant feeding expectations, goals and building rapport. A home visit was conducted up to two weeks post-delivery to ensure breastfeeding was established and for the participant to discuss any concerns. Follow up phone calls were conducted as required, to address the raised concerns of the individual participants.”

#### 2.2.5. Outcome Measures

Data collection followed many of the same procedures as had been previously employed in the Women and their children (WATCH) Study and further details are published elsewhere [29].

#### 2.2.6. Weight and BMI

Weight was measured, to the nearest 50 grams, in indoor clothing without shoes using AND™ FV-150 K electronic weighing scales (A & D Mercury Pty Ltd., Thebarton, South Australia), which were calibrated annually according to hospital protocol. Height was measured without shoes, to the nearest millimeter, on a wall mounted Seca stadiometer (Seca Deutschland, Hamburg, Germany).

#### 2.2.7. Biomarkers

Blood collection and analyses were outsourced to the Hunter Area Pathology Service, a National Association of Testing Authorities (NATA)-accredited laboratory. Samples were obtained at 35 weeks gestation (visit 2), three months postpartum (visit 3) and six months postpartum (visit 4). Fasting blood samples were analysed for fasting glucose (mmol/L), fasting insulin (mIU/L), glycosylated haemoglobin (HbA1c, %), lipids (total cholesterol, LDL, HDL, and triglycerides, in mmol/L), and C-reactive protein (mg/L). Homeostatic model assessment (HOMA-IR) was estimated: (glucose × insulin)/22.5.

#### 2.2.8. Breastfeeding

Infant feeding data were obtained by the study dietitian at the three and six month follow up. The Infant Feeding Recall questionnaire was used to collect information on breastfeeding initiation, duration and exclusivity. The Current Feeding Practices questionnaire was also used to record the infant’s breastfeeding and formula intake and whether the child received any of the following within the previous 24 h: vitamin or mineral supplements; medicine; plain water; sweetened or flavoured water (for example, cordials and soft drinks); fruit juice; tea or infusion; canned, powdered, or fresh milk; solid or semi-solid foods; oral rehydration salts; and other foods or fluids.

#### 2.2.9. Further Data Collection

A 13-item questionnaire was administered in study visit 1 to determine medical history and intake of prescribed and/or non-prescribed medication including supplements. Education level, socio economic advantage and disadvantage using Socio-Economic Indexes for Areas (SEIFA) index, income and marital status were assessed by asking another six questions, modelled on the Australian Longitudinal Study of Women’s Health surveys (http://alswh.org.au/). The Index of Relative Socioeconomic Advantage and Disadvantage (IRSAD) was used to provide information on social and economic status of households based on area. The IRSAD is scored one to ten with a low score indicating greater disadvantage and a higher score indicating greater advantage.

### 2.3. Statistical Analyses

Statistical analyses were performed using Intercooled Stata, version 11 (StataCorp LP, College Station, Texas, USA) with significance set at α = 0.05. Normality tests were conducted to determine the distribution of data. One-way ANOVA, Kruskal-Wallis, Chi^2^, and Fisher’s exact tests were used to determine differences by intervention groups. Comparisons were also made between those who withdrew and those who remained in the study. Intention to treat analysis was not conducted as this was a pilot feasibility study with small numbers in each group.

## 3. Results

Figure 1 summarises participant recruitment, randomisation and follow-up. Thirty-six women consented (22 overweight, 14 obese), and 12 were randomised to each group, with two were lost to follow-up before the first visit. At six months postpartum, 25 of participants completed the study. Women who were lost to follow-up reported being too busy (*n* = 1), were not able to be contacted by the research team (*n* = 4), had a preterm delivery and chose to discontinue participation (*n* = 1), moved interstate (*n* = 1), or changed hospitals during antenatal care (*n* = 1).

Table 1 summarises the baseline characteristics and birth data for all participants and by group allocation. The mean age for the study population was 31 years and 85% of participants were having their first birth. Just over half the babies were female (58%) and the mean birth weight was 3.7 kg. There were no significant differences in baseline socio-demographic variables between those who withdrew and those who remained in the study, though the study was underpowered to detect any differences (data not shown).

**Table 1:** Baseline characteristics and birth data for pilot randomised controlled trial participants.

Participant Characteristic	All (*n* = 34)	Diet (*n* = 11)	Diet + Lactation Support (*n* = 11)	Control (*n* = 12)	*p*-Value
Age (years)	30.9 ± 6.0	29.5 ± 7.8	31.6 ± 5.1	31.3 ± 5.6	0.27
Height (cm)	165.4 ± 6.2	165.1 ± 6.5	166.8 ± 5.7	164.4 ± 6.5	0.44
Born in Australia, *n* (%)	33/34 (97)	10/11 (91)	11/11 (100)	12/12 (100)	0.65
Married or *de facto,* *n* (%)	25/31 (81)	7/10 (70)	8/9 (89)	10/12 (83)	0.63
Education ≥ year 12, *n* (%)	26/31 (84)	9/10 (90)	8/9 (89)	9/12 (75)	0.79
IRSAD ≥ 5, *n* (%)	23/32 (72)	7/10 (70)	9/10 (90)	7/12 (58)	0.22
Smoking, *n* (%)	4/34 (12)	2/11 (18)	1/11 (9)	1/12 (8)	1.00
Multiparous, *n* (%)	5/34 (15)	1/11 (9)	2/11 (18)	2/12 (17)	0.39
Gestational diabetes, *n* (%)	3/34 (9)	2/11 (18)	1/11 (9)	0/12 (0)	0.76
Infant sex, male *n* (%)	14/33 (42)	3/11 (27)	6/10 (60)	5/12 (42)	0.23
Birth weight (kg)	3.7 (3.3, 4.0)	3.9 (3.7, 4.1)	3.5 (3.1, 3.7)	3.5 (3.2, 4.0)	0.19

Mean ± SD for normally distributed continuous data or median (25th–75th percentile) for non-normally distributed data.

Table 2 summarises the pregnancy and postpartum weight data for the randomised controlled trial participants. Self-reported mean pre-pregnancy weight was 80.5 kg and the median BMI was 28.8 kg/m^2^. By 35 weeks gestation the women had gained on average 13.6 ± 6.6 kg (mean ± SD). This is considerably higher than the Institute of Medicine’s recommendations of 5–11.5 kg for women with a BMI of 25 or more [30], especially considering the final weight measurement was at 35 rather than 40 weeks gestation. Mean gestational weight gain in the dietary intervention group at 35 weeks gestation was on average 5–6 kg lower, however, this was not significant (*p* = 0.06) and may have occurred by chance. At three months postpartum weight retention was not significantly different between groups, however those in the dietary intervention groups (with or without lactation support) retained less weight compared to the control group. At three months postpartum, control participants commenced the dietary intervention and there was less difference in weight retention at six months postpartum. There was a trend for fewer women in the dietary intervention group to retain 5 kg or more than their pre-pregnancy weight at six months after birth, compared to the control group (*p* = 0.06).

Table 3 summarises the fasting biochemical data including glycaemic markers, lipids and the inflammatory biomarker CRP. There were no significant differences by group allocation.

All participants initiated breastfeeding which was to be expected on the basis of the inclusion criteria. At three months postpartum, the majority of participants had continued breastfeeding (74%). The dietary intervention with lactation support group had the highest rates of breastfeeding compared to the dietary intervention alone and the control groups though the differences were not significant.

**Table 2:** Weight data for pilot randomised controlled trial participants.

Time	Variable	All (*n* = 34)	Diet (*n* = 11)	Diet + Lactation Support (*n* = 11)	Control (*n* = 12)
Pre-pregnancy	Weight (kg)	80.5 ± 12.0	81.5 ± 15.1	81.6 ± 9.8	78.5 ± 11.3
	BMI (kg/m^2^)	28.8 (25.4, 32.3)	27.7 (25.1, 33.7)	29.4 (25.5, 32.3)	28.9 (25.2, 32.9)
Pregnancy	Weight gain (kg) ^a^	13.6 ± 6.6	9.8 ± 4.5	15.3 ± 7.2	16.0 ± 6.7
Postpartum—3 months	Weight	84.9 ± 13.7	84.2 ± 14.0	85.9 ± 14.3	84.8 ± 14.3
	BMI	30.8 ± 4.2	30.7 ± 4.1	30.6 ± 5.4	31.1 ± 3.9
	Weight retention (kg) ^b^	4.6 ± 7.4	0.91 ± 7.03	4.4 ± 7.6	7.7 ± 6.8
Postpartum—6 months	Weight	85.3 ± 13.0	84.2 ± 14.7	89.9 ± 11.3	82.7 ± 13.1
	BMI	30.7 ± 3.7	30.6 ± 4.3	31.2 ± 4.4	30.3 ± 3.0
	Weight retention	3.3 ± 4.0	0.8 ± 7.2	5.6 ± 8.8	5.9 ± 4.9
	Weight ≤ pre-pregnancy, *n* (%)	5/25 (20)	3/9 (33)	2/7 (29)	1/9 (11)
	Weight retention ≥ 5 kg, *n* (%)	10/25 (40)	1/9 (11)	4/7 (57)	5/9 (56)

Mean ± SD for normally distributed continuous data or median (25th–75th percentile) for non-normally distributed data. There were no significant differences found between study groups. ^a^ Weight gain = Weight measured at 35 weeks gestation—self-reported pre-pregnancy weight; ^b^ Weight retention = Weight at visit—self-reported pre-pregnancy weight.

**Table 3:** Fasting biochemical data for pilot randomised controlled trial participants.

Time	Variable	All (*n* = 34)	Diet (*n* = 11)	Diet + Lactation Support (*n* = 11)	Control (*n* = 12)	*p*-Value
Pregnancy	Glucose (mmol/L)	4.2 ± 0.4	4.3 ± 0.5	4.2 ± 0.4	4.2 ± 0.2	0.99
	Insulin (mIU/L)	9.1 ± 3.6	8.3 ± 3.0	8.8 ± 4.4	10.1 ± 3.4	0.46
	HbA1c (%)	5.3 (5.2, 5.5)	5.3 (5.1, 5.5)	5.3 (5.1, 5.5)	5.4 (5.2, 5.6)	0.48
	HOMA-IR ^a^	1.7 ± 0.8	1.6 ± 0.7	1.7 ± 0.9	1.9 ± 0.7	0.62
	Total cholesterol (mmol/L)	6.8 ± 1.2	6.7 ± 1.0	6.6 ± 1.2	7.1 ± 1.4	0.77
	Triglycerides (mmol/L)	2.3 ± 0.7	2.0 ± 0.4	2.3 ± 0.8	2.6 ± 0.7	0.11
	LDL-C (mmol/L)	3.9 ± 1.1	4.0 ± 0.7	3.6 ± 1.1	4.1 ± 1.3	0.56
	HDL-C (mmol/L)	1.9 ± 0.5	1.9 ± 0.6	1.9 ± 0.5	1.9 ± 0.4	0.95
	Total/HDL-C	3.8 ± 0.8	3.7 ± 0.8	3.7 ± 1.0	3.9 ± 0.7	0.78
	CRP (mg/L)	7.3 (4.8, 12.9)	11.1 (6.3, 13.7)	6.7 (3.0, 14.6)	7.3 (5.2, 9.5)	0.91
Postpartum—3 months	Glucose	4.6 ± 0.5	4.5 ± 0.5	4.4 ± 0.4	4.7 ± 0.5	0.58
	Insulin	5.5 (3.3, 6.8)	5.2 (4.6, 10.3)	5.5 (3.2, 6.4)	5.4 (3.2, 6.8)	0.61
	HbA1c	5.3 ± 0.2	5.3 ± 0.2	5.3 ± 0.2	5.5 ± 0.4	0.39
	HOMA-IR	1.0 (0.7, 1.4)	1.0 (0.8, 2.2)	1.2 (0.6, 1.4)	1.1 (0.6, 1.4)	0.69
	Total cholesterol	5.0 ± 1.0	5.2 ± 1.3	4.4 ± 0.6	5.2 ± 0.7	0.31
	Triglycerides	1.0 ± 0.6	1.2 ± 0.8	0.7 ± 0.2	1.0 ± 0.6	0.25
	LDL-C	3.0 ± 0.7	2.9 ± 0.7	2.6 ± 0.6	3.1 ± 0.7	0.36
	HDL-C	1.6 ± 0.5	1.7 ± 0.7	1.5 ± 0.3	1.6 ± 0.4	0.84
	Total/HDL-C	3.4 (2.5, 3.6)	3.5 (2.7, 3.8)	3.0 (2.9, 3.4)	3.4 (2.5, 4.4)	0.61
	CRP	6.2 ± 4.9	7.8 ± 6.4	5.6 ± 3.5	5.3 ± 4.1	0.56
Postpartum—6 months	Glucose	4.5 ± 0.4	4.5 ± 0.5	4.5 ± 0.4	4.6 ± 0.4	0.91
	Insulin	5.4 (3.2, 6.1)	5.8 (4.5, 10.2)	3.5 (2.7, 9.3)	5.4 (2.9, 6.0)	0.55
	HbA1c	5.3 ± 0.3	5.3 ± 0.4	5.3 ± 0.2	5.3 ± 0.3	0.88
	HOMA-IR	1.1 (0.6, 1.4)	1.1 (0.87, 1.83)	0.7 (0.5, 2.0)	1.0 (0.6, 1.3)	0.65
	Total cholesterol	4.8 ± 1.2	4.7 ± 1.6	4.8 ± 1.0	4.9 ± 1.0	0.95
	Triglycerides	0.9 (0.5, 1.2)	1.0 (0.5, 1.4)	0.8 (0.7, 1.1)	0.9 (0.5, 1.4)	0.70
	LDL-C	2.9 ± 1.0	2.7 ± 1.0	3.0 ± 1.2	3.0 ± 1.0	0.83
	HDL-C	1.3 (1.1, 1.8)	1.2 (1.1, 1.9)	1.3 (1.3, 1.3)	1.5 (1.1, 1.7)	0.94
	Total/HDL-C	3.4 (2.6, 4.3)	3.4 (2.7, 4.3)	3.7 (3.0, 4.3)	3.1 (3.0, 4.4)	0.92
	CRP	3.5 (3.0, 6.1)	3.3 (2.0, 6.1)	5.2 (2.5, 6.6)	3.1 (3.0, 4.4)	0.98

Mean ± SD for normally distributed continuous data or median (25th–75th percentile) for non-normally distributed data. ^a^ HOMA-IR, Homeostatic Model Assessment of Insulin Resistance: (glucose mmol/L × insulin mIU/L)/22.5.

### Trial Evaluation

Fourteen of the participants who completed the study returned the evaluation questionnaire for the TEMplate™ program. Of those who returned the questionnaire, six agreed that the TEMplate™ program was easy to understand. On completion of the TEMplate™ program, the majority of participants reported that they now weigh themselves (*n* = 10), keep a record of what they eat (*n* = 5), try to be more active (*n* = 14), and plan (*n* = 9) and cook healthier meals (*n* = 13). Two of the participants found the TEMplate™ program too time consuming, and six found it difficult to use the program whilst also caring for a new baby. Additional barriers that were reported included time constraints (*n* = 7), and tiredness (*n* = 6). Two of the participants also suggested they would prefer face-to-face contact with a dietitian (*n* = 2) during the program.

## 4. Discussion

This pilot RCT examined the feasibility of a program that included dietary intervention and breastfeeding support for overweight and obese pregnant women, with the aim of reducing postpartum weight retention and enhancing rates of breastfeeding. As a pilot study, it was not intended to be adequately powered to show significant between group differences in outcomes of interest: weight, biomarkers, and breastfeeding. However, the results indicate that the approach is feasible and acceptable to pregnant women attending an antenatal clinic and that the methodology, including the collection of blood for biomarker assessment, and could be adapted based on qualitative feedback to a larger, adequately powered RCT.

The use of paid, dedicated research midwives to undertake the recruitment of women in an antenatal clinic setting would be an improvement on the recruitment strategy used in this pilot, given the known difficulties in recruitment in this population. Retention was at 69%, which is not ideal. However, the issues identified such as difficulty attending the primary measurement site will allow for better retention in the subsequent RCT. Women were lost to follow up for a number of reasons, in particular due to the burden of attending face to face visits. The use of a flexible delivery model, such as a web-based intervention strategy, combined with home visits for follow-up, should be considered to overcome this barrier.

Research shows the postpartum period to be associated with many adjustments for a mother, including increased time constraints, a change in priorities and child care concerns [31,32]. These adjustments can make it difficult for women to achieve a healthy lifestyle [31,32] and have been reported to contribute to the lack of success in postpartum weight loss interventions. High drop-out (up to 40%) and low attendance rates [33,34,35], led researchers to conduct interviews on participants from the Active Mothers Postpartum (AMP) study to further investigate lack of participation in this cohort of women [32]. Results indicate numerous barriers to achieving a healthy lifestyle at this life-stage, including: lack of time in a busy schedule; health of the family as first priority; lack of social support; lack of child care during the intervention and/or education; and location distant from the study centre [32]. This pilot study attempted to address a number of these reported issues. Women were provided with a self-management program to complete in their own time, which increased flexibility and eliminated the need for child care. Phone calls and home visits were offered for women who were unable to visit the hospital due to geographical location or who had child care issues. Additionally, in the current study the weight management education was provided during an antenatal visit towards the end of pregnancy to help prepare and organise the women for the postpartum period. Despite anticipating the known barriers in the literature to a weight loss program, there were substantial withdrawals from the program, however they predominantly occurred during the antenatal period.

Results from the evaluation suggest the majority of participants reported improved awareness of their eating and exercise behaviours as a result of the intervention. The participants indicated that they were able to record intake, monitor weight, and increase everyday exercise, plan meals and make healthier food choices using the provided program materials. Still, half of the women indicated they found it difficult to complete the program with a new baby, despite this being an intervention which they could complete in their own home. From this feedback we can deduce that any intervention in this life-stage must be goal-orientated, include self-monitoring, and be home-based, while also being quick and easy. Using web-based or mobile device technologies may be one way to enhance access within the home, to address the time and travel barriers expressed in the evaluations.

All the participants in this pilot study initiated breastfeeding, but the duration of breastfeeding was greatest in the group with additional lactation support, though small numbers meant this was not statistically significant. The provision of IBCLC support appeared to be both feasible and acceptable to overweight and obese mothers. Suboptimal breastfeeding initiation and duration rates have been frequently cited for women with a high BMI [15,25,36,37,38,39]. In a systematic review of 15 observational studies the relationship between maternal overweight and obesity and breastfeeding initiation and duration identified the rates for any breastfeeding at six months in overweight women were 17% to 52% while rates for obese women were 17% to 37% compared to normal weight women (29% to 57%) [40].

For pregnancy and postpartum metabolic markers the direction of the results was in the intended direction, indicating a potential improvement in metabolic profile due to the intervention, although not statistically significant. The pilot study was not powered to show significance. Further research in this area is warranted.

Research to manage postpartum weight retention is of increasing interest. There have been a number of lifestyle RCTs aimed at reducing postpartum weight retention or increasing weight loss [33,34,35,41,42,43,44]. Two of these studies had lower weight retention for both intervention and control groups compared to the current study although only one of these was significant (Control *versus* intervention: 1.0 and 1.8, *p* = 0.42; 5.1 and 2.3, *p* < 0.001) [42,43]. However, none of these studies include a breastfeeding support commencing in pregnancy; a novel component of our trial. There are a number of important methodological differences between the current study and RCTs conducted to date. The strength of the current study is the recruitment of women in the antenatal period to prepare them to achieve postpartum weight loss. Early recruitment of participants may have the added advantage of preventing gestational weight gain, although our study participants generally gained more than current recommendations [27]. Similarly, the current study uses self-reported pre-pregnancy weight, combined with weight at initial visit as objective markers of gestational weight gain and weight retention. The current study primarily focused on dietary intervention. Finally focusing on overweight and obese women targets those who may have difficulty initiating breastfeeding while supporting longer breastfeeding duration.

Our pilot study participants in the dietary intervention only group retained less weight at both three and six months postpartum compared to the two other RCTs reporting weight retention as their primary outcome [42,43]. Kinnunen *et al.* (2007) intervened during the postpartum period focusing on lifestyle change to reduce weight retention [43]. Huang, Yeh and Tsai (2011) provided weight management education during pregnancy, similar to our pilot study, however, the aim of their intervention was to limit gestational weight gain, rather than prepare the participants solely for weight loss in the postpartum period [42]. This makes it difficult to determine the postpartum impact of this intervention because gestational weight gain is a significant determinant of weight retention [45,46].

This pilot study had a number of limitations that need to be acknowledged. The primary limitation is the small sample size, making it difficult to detect significant differences in outcome variables and limiting the applicability of the study to the general population. However, as this was a feasibility study, it is more important to look at the recruitment and retention information and the evaluation data. In addition to this, the retention rate was suboptimal suggesting the need for further consideration with this population and their unique stage of life. To improve the study sample size and retention rates, regular contact with a health professional and incentives to complete the study could be considered. Additionally, providing flexible study visits to suit mothers’ postpartum lifestyles may require home visits, regular phone calls, and internet support.

## 5. Conclusions

Overweight and obesity in women is an independent risk factor for lifestyle disease such as diabetes and heart disease [47,48]. Pregnancy can further impact this due to gestational weight gain, postpartum weight retention and lower rates of successful breastfeeding initiation and duration. Lifestyle changes, such as healthy eating and exercise, which are used in traditional weight loss programs, may be problematic for women with infants because of time constraints and changes in priorities. Despite postpartum weight retention being common and a relevant concern for women, there are currently no programs or strategies routinely offered to assist women to achieve a healthy weight after birth. The current study provides evidence to support the feasibility and preliminary efficacy of providing overweight and obese women with targeted dietary advice and breastfeeding support to improve weight, metabolic, and breastfeeding outcomes. An adequately powered RCT is now required to determine the true effect, if any, of these interventions and the costs involved in implementing those that are effective on a larger scale.
